# CLK2 in GABAergic neurons is critical in regulating energy balance and anxiety-like behavior in a gender-specific fashion

**DOI:** 10.3389/fendo.2023.1172835

**Published:** 2023-08-10

**Authors:** Sónia Norberto, Heloisa Balan Assalin, Dioze Guadagnini, Natália Tobar, Patrícia Aline Boer, Min-Cheol Kang, Mario Jose Abdalla Saad, Young-Bum Kim, Patricia Oliveira Prada

**Affiliations:** ^1^ Department of Internal Medicine, School of Medical Science, University of Campinas (UNICAMP), Campinas, SP, Brazil; ^2^ Department of Radiology, University of Campinas (UNICAMP), Campinas, SP, Brazil; ^3^ Department of Internal Medicine, Fetal Programming Laboratory, School of Medical Science, University of Campinas (UNICAMP), Campinas, SP, Brazil; ^4^ Division of Endocrinology, Diabetes and Metabolism, Department of Medicine, Beth Israel Deaconess Medical Center, Harvard Medical School, Boston, MA, United States; ^5^ Research Group of Food Processing, Korea Food Research Instute, Jeollabuk-do, Wanju, Republic of Korea; ^6^ School of Applied Sciences, University of Campinas (UNICAMP), Limeira, SP, Brazil; ^7^ Max-Planck Institute for Metabolism Research, Köln, Germany

**Keywords:** Cdc2-like kinase, CLK2, GABAergic neurons, hypothalamus, glucose intolerance, food intake energy expenditure, anxiety-like behavior

## Abstract

**Introduction:**

Cdc2-like kinase (CLK2) is a member of CLK kinases expressed in hypothalamic neurons and is activated in response to refeeding, leptin, or insulin. Diet-induced obesity and leptin receptor-deficient *db/db* mice lack CLK2 signal in the hypothalamic neurons. The neurotransmiter gamma-aminobutyric acid (GABA) is among the most prevalent in the central nervous system (CNS), particularly in the hypothalamus. Given the abundance of GABA-expressing neurons and their potential influence on regulating energy and behavioral homeostasis, we aimed to explore whether the deletion of CLK2 in GABAergic neurons alters energy homeostasis and behavioral and cognitive functions in both genders of mice lacking CLK2 in Vgat-expressing neurons (Vgat-Cre; Clk2^loxP/loxP^) on chow diet.

**Methods:**

We generated mice lacking Clk2 in Vgat-expressing neurons (Vgat-Cre; Clk2^loxP/loxP^) by mating Clk2^loxP/loxP^ mice with Vgat-IRES-Cre transgenic mice and employed behavior, and physiological tests, and molecular approaches to investigate energy metabolism and behavior phenotype of both genders.

**Results and discussion:**

We showed that deletion of CLK2 in GABAergic neurons increased adiposity and food intake in females. The mechanisms behind these effects were likely due, at least in part, to hypothalamic insulin resistance and upregulation of hypothalamic Npy and Agrp expression. Besides normal insulin and pyruvate sensitivity, Vgat-Cre; Clk2^loxP/loxP^ females were glucose intolerant. Male Vgat-Cre; Clk2^loxP/loxP^ mice showed an increased energy expenditure (EE). Risen EE may account for avoiding weight and fat mass gain in male Vgat-Cre; Clk2^loxP/loxP^ mice. Vgat-Cre; Clk2^loxP/loxP^ mice had no alteration in cognition or memory functions in both genders. Interestingly, deleting CLK2 in GABAergic neurons changed anxiety-like behavior only in females, not males. These findings suggest that CLK2 in GABAergic neurons is critical in regulating energy balance and anxiety-like behavior in a gender-specific fashion and could be a molecular therapeutic target for combating obesity associated with psychological disorders in females.

## 1 Introduction

The worldwide prevalence of obesity has increased in recent decades, affecting the quality and expectancy of life (1). It is the leading risk factor for cardiometabolic diseases such as type 2 diabetes and coronary heart disease (1–4). Obesity is also associated with mental illnesses such as anxiety and psychiatric disorders (5–7).

Excessive adiposity in adipose tissues defines overweight and obesity (8). The etiopathogenesis of obesity involves primarily positive energy intake and lower energy expenditure, as well as the environment, socioeconomic conditions, behavior, and genotype-phenotype interactions (9–13). Thus, it is essential to understand the mechanisms contributing to developing and maintaining obesity and its complications and identify potential targets for therapeutic interventions.

The hypothalamus controls energy homeostasis by integrating hormonal, nutritional, and neural signals (14–16). The arcuate nucleus of the hypothalamus (ARC) holds neurons expressing proopiomelanocortin (POMC), which suppresses feeding. Conversely, it includes agouti-related peptide (AgRP) neurons, which increases food intake (14–17). Fasting raises AgRP neuronal excitability, and refeeding inhibits AgRP activation (18–20). Upon activation, AgRP neurons release NPY and the inhibitory neurotransmitter γ-aminobutyric acid (GABA), which induces feeding (21, 22). Therefore, AgRP neurons are mostly GABAergic cells. The release of GABA by AgRP neurons inhibits POMC neurons contributing to the orexigenic effect. Activating AgRP projections to PVH (paraventricular nucleus of the hypothalamus), which expresses corticotropin-releasing hormone (CRH) neurons, increases the release of CRH (23, 24). CRH has anorectic effects and thermogenic actions, further regulating energy balance (23, 25).

Insulin receptors (IR) are abundant in the central nervous system (CNS) and expressed in the ARC’s AgRP and POMC neurons (26). Insulin acts via IR, activating the protein kinase B (AKT) pathway, and the outcome will depend on the cell type (27). In AgRP neurons, insulin decreases NPY and AgRP release, which blocks feeding (27). In POMC neurons, insulin may activate or inhibit their firing. The activation of IR in a specific population of POMC increases these neurons firing, contributing to decrease feeding (28).

The Cdc2-like kinase (CLK2) is a member of CLK kinases (LAMMER kinases) identified as a dual-specificity protein kinase, i.e., the protein can phosphorylate its substrates on serine/threonine and tyrosine (29). Despite the limited understanding of CLK targets, function, and regulation in the biological system, CLK2 has a role in energy metabolism. Refeeding activates CLK2 (30–32). The activation of CLK2 leads to the phosphorylation of the SR domain on PGC-1α in the liver, repressing hepatic gluconeogenesis and glucose production (30). Through liver-specific CLK2 knockout mice, CLK2 regulates hepatic fat metabolism, fatty acid oxidation, and ketogenesis during fasting in diet-induced obesity (DIO) mice (33). Mice lacking CLK2 in adipose tissue (AT) exhibited decreased energy expenditure showing lower Ucp1 levels in brown adipose tissue (BAT) in mice under a high-fat diet (HFD) intermittent fasting regimen (34).

In our previous study (32), CLK2 was expressed in hypothalamic neurons, not astroglial cells, and distributed in several brain structures (32). We also showed that hypothalamic CLK2 is phosphorylated at threonine 343 in response to refeeding, leptin, and insulin (32). This result was similar to what occurred in the liver in previous studies involving hepatic CLK2 (30, 31). In addition, we observed that obese mice (*db/db* and DIO mice) had an impairment of this regulation in the hypothalamus. Pharmacological inhibition and the knockdown of hypothalamic Clk2 led to obesity due to hyperphagia and lower energy expenditure (32). Contrarily, overexpressing Clk2 by adenovirus in the mediobasal hypothalamus of obese mice partly restored energy homeostasis (32). Hence, our previous study suggested that CLK2 integrates the insulin and leptin pathways in the hypothalamus, and CLK2 regulation affects energy homeostasis (32).

Neurons expressing γ-aminobutyric acid (GABAergic) are abundant in the CNS (35–37), turning GABA into useful markers to study the effect of gene deletion or overexpression, resulting in a significant physiological impact. GABA neurotransmitter is dominant in the hypothalamus, emphasizing the importance of inhibitory circuits in this region (38). Specific ablation of insulin receptor (IR) in GABA-expressing neurons increased body weight and adiposity compared to control animals (39). Ablation of LEPR also in GABA-expressing neurons triggered severe obesity (39–41). In contrast, mice with specific LEPR deletion in GLU neurons exhibited minor differences in weight gain and adipose mass (37). These studies suggest that GABAergic neurons significantly affect body weight regulation (39). In our first study, we observed that CLK2 was expressed in hypothalamic neurons, and chronic knockdown of CLK2 in the hypothalamus impaired insulin and leptin action, and the animal ate more (32). Given the abundance of neurons expressing GABA in the hypothalamus and their role in controlling feeding and anxiety behavior, we aimed to investigate the impact on fuel metabolism and behavior changes of CLK2 deletion in GABAergic neurons, focused on the hypothalamic area underlining possible gender differences in the phenotype.

## 2 Materials and methods

### 2.1 Animal care

The Ethics Committee for Animal Experimentation at the University of Campinas (UNICAMP), São Paulo, Brazil, approved the experimental protocol used in this study (CEUA 5103-1/2018, CEUA 5102-1/2018 and CIBio 23/2018). The Committee mentioned above follows the National Institute of Health Guidelines for using experimental animals. Mice were at least four weeks of age at the beginning of the study, and they were kept on a 12 h light-dark cycle with lights on at 6:00 AM and lights off at 6:00 PM, at 22-24°C. Unless otherwise indicated, mice had access to a normocaloric chow diet (NCD, 339 kcal/100g; Nuvilab^®^ CR-1, Nuvital, PR-Brasil) and water supplied *ad libitum*.

### 2.2 Generation of GABAergic neuron-specific CLK2 knockout mice

Mice bearing a loxP-flanked Clk2 allele (Clk2^loxP/loxP^) were obtained by Pere Puigserver (Dana-Farber Cancer Institute, Boston, MA) (33). Mice lacking Clk2 in Vgat-expressing neurons (Vgat-Cre; Clk2^loxP/loxP^) were generated by mating Clk2^loxP/loxP^ mice with Vgat-IRES-Cre transgenic mice (22, 37) (gift from Dr. Brad Lowell, Beth Israel Deaconess Medical Center, Boston, MA). All mice we studied are mixed backgrounds with 129 and C57BL/6 mice strains. Previously, targeting constructs were prepared using mouse 129 BAC genomic clones to generate Vgat-IRES-Cre mice (22, 37, 42) and genomic DNA fragments from the C57BL/6 mouse strain to generate Clk2^loxP/loxP^ mice (33), and electroporated into ES cells. We used littermate Clk2^loxP/loxP^ mice as controls. Our breeding strategy was the following: male and female Clk2^loxP/loxP^ mice and male and female Vgat-IRES-Cre crossed with male or female C57BL/6 mice to generate more Clk2^loxP/+^ mice and Vgat-Cre/+ mice to have enough mice to start a colony. Next, Clk2^loxP/+^ mice crossed with Vgat-IRES-Cre to generate Vgat-Cre/+; Clk2^loxP/+^ mice. By mating Clk2^loxP/+^ with Vgat-Cre/+; Clk2^loxP/+^, we generated our main interest group as Vgat-Cre+/-; Clk2^loxP/loxP^ mice. By crossing Vgat-Cre+/-; Clk2^loxP/loxP^ mice amongst each other, we obtained more Clk2^loxP/loxP^ mice to use as a control and more Vgat-Cre; Clk2^loxP/loxP^ mice. We used conventional PCR to genotype all mice used in this study. Briefly, we amplified genomic DNA extracted from the tail using REDExtract-N-Amp PCRTM ReadyMixTM (Sigma-Aldrich Co. LLC, Brazil). We list all primers in **Supplementary Table 1**. PCR products were size-separated by agarose gel electrophoresis and visualized with GelRed 1:500 (GelRed^®^Nucleic Acid Stain, Biotium), capturing the images in the Gel Doc System (Bio-Rad Universal Hood III, California, USA). We used Clk2^loxP/loxP^ mice as a control group.

### 2.3 Body mass and body composition measurements

Male and female mice were weighed weekly from 4 weeks to 26-28 weeks. Mice were anesthetized with a mixture of ketamine (100 mg/kg of BW) and xylazine (10 mg/kg of BW), and the body composition (whole-body fat and lean masses) was assessed using Dual-energy X-ray Absorptiometry (DEXA) system (Discovery Wi 83901 QDR Series; Hologic Apex Software v13.3:5, Hologic Inc) at 22-25 weeks. Bone mass was not considered in lean mass values. In addition to DEXA, the adipose tissue pads were isolated and weighed at the end of the study.

### 2.4 Food intake

For average daily food intake measurements, we recorded food intake for five consecutive days. Before starting the measurements, mice (16-18 weeks) were individually housed in metabolic cages and maintained for 24 h to adapt to the new cages. Average daily food intake was calculated by subtracting the remained food in a metabolic cage from the delivered food for each mouse.

### 2.5 Energy expenditure

The energy expenditure was assessed by indirect calorimetry using an Oxylet M3 System (PanLab, Harvard Apparatus). Mice at 16-18-week-old were housed individually and acclimated in the apparatus boxes for 24 h prior to the measurements of oxygen consumption (VO2), carbon dioxide production (VCO2), heat production (HEAT), and respiratory exchange ratio (RER) for 24 h.

### 2.6 Glucose, insulin, and pyruvate tolerance tests

For the glucose tolerance test (GTT), we injected glucose (1.0 g/kg of body weight (BW) intraperitoneal (IP) in overnight (7:00 PM to 8:00 AM) fasted mice (8 weeks). Blood glucose was determined before the experiment and at 15, 30, 45, 60, and 120 min after an IP injection of glucose. For the insulin tolerance test (ITT), mice were 9 weeks and fasted for 6 h (8:00 AM to 2:00 PM). Blood glucose was measured before and at 5, 10, 15, 20, 25, and 30 min after an IP injection of insulin (1.0 IU/kg of BW; Human recombinant insulin; Eli Lilly) (43). The pyruvate tolerance test (PTT) was performed to measure hepatic gluconeogenesis indirectly. Overnight (7:00 PM to 8:00 AM) fasted mice at 10 weeks were injected with sodium pyruvate (1.5 g/kg of BW; ReagentPlus, Sigma-Aldrich). Blood glucose was measured before and at 15, 30, 45, 60, 90, and 120 min after the IP injection of sodium pyruvate (32). The area under the curve (AUC) for blood glucose was calculated for GTT and PTT. Blood glucose decay constant (kITT) was calculated from the slope of the least square analysis of the blood glucose concentrations during the linear phase of the decay and was used to estimate insulin sensitivity after ITT (44, 45).

### 2.7 Blood parameter measurements

For GTT, ITT, and PTT tests, blood glucose levels were measured from the tail using an automatic glucometer (Accu-Chek Active, Roche). Serum insulin levels from blood samples collected via the tail during GTT, fasting serum insulin, and fasting leptin levels from blood samples collected at final of the experiments were measured by ELISA (Rat/mouse Insulin ELISA Kit, EZRMI-13K, and Mouse Leptin ELISA Kit, EZML-82K, Millipore).

### 2.8 Hypothalamic insulin responsiveness

To determine hypothalamic insulin sensitivity, both genders Vgat-Cre; Clk2^loxP/loxP,^and Clk2^loxP/loxP^ mice, were housed individually at 25-28 weeks. Intracerebroventricular (ICV) cannula implantation was performed following the Atlas Paxinos’ coordinates: anterior/posterior: -0.5 mm, lateral: -1.3 mm, and dorsal/ventral: -2.2 mm to reach a lateral ventricle as described before (32, 46, 47). After 2-3 days of recovery, the cannula positions were confirmed by ICV injection of angiotensin II, which induces a dipsogenic effect (48). Animals that did not reach this criterion were excluded from the experiments. Before the experiments, mice were fasted overnight (8:00 PM to 9:00 AM). At 9:00 AM, we injected via ICV insulin (0.2 μg/μl; Human recombinant insulin, Eli Lilly) or saline (0.9% NaCl) solutions and recorded food intake after 4, 8, 12, and 24 h. For the dissection of the hypothalamus, we removed and placed the ventral view of the brain on a Petri dish with saline. Using forceps and a razor, we dissected the hypothalamus avoiding the optic chiasm and optic tract and removing hypothalamic surrounding membranes and vessels (49). The hypothalamic sample was immediately placed inside a tube and frozen at -80 C in liquid nitrogen until protein extraction and Western Blotting analysis to determine p-AKT phosphorylation in response to insulin.

### 2.9 Protein extraction and western blotting

We injected overnight (8:00 PM to 9:00 AM) fasted mice with insulin (0.2 μg/μl) or saline (0.9% NaCl) via ICV to determine p-AKT phosphorylation in response to insulin. After 15 minutes of ICV, we euthanized mice for the hypothalami dissection. Brown adipose tissue (BAT) and liver were also obtained. Samples were immediately frozen in liquid nitrogen until protein extraction and Western Blotting. Protein extract from the hypothalamus, BAT, or liver were obtained using the Lysis buffer described before (32, 46, 47). Briefly, the lysates (60 μg protein) were separated using SDS-PAGE electrophoresis and transferred to nitrocellulose membranes. The membranes were incubated overnight with primary antibody UCP-1 (M-17, #sc-6529 Santa Cruz Biotechnology, Dallas, TX, USA), pAKT Ser 473 (#9271S) or total AKT (Akt (pan) (11E7), #4685S), both from Cell Signaling Technology (Boston, MA, USA), using samples collected after ICV injection of insulin. β-Actin (#4967, Cell Signaling Technology, Boston, MA, USA) was used as an endogenous control in all membranes. After the incubation with respective secondary antibodies, bound antibodies were visualized using the chemiluminescence western blotting substrate (SuperSignal West Pico Chemiluminescent Substrate, Thermo Scientific). The images were captured by Gel Doc System (Bio-Rad Universal Hood III, CA, USA), and their software (Image Lab, version 6.0.1) performed the quantifications used in this paper.

### 2.10 RNA extraction and real-time PCR

Total RNA from interscapular BAT and hypothalamus were extracted using the RNeasy Mini kit (#74106; Qiagen Inc, CA, USA). We followed a previous protocol (32) for the cDNA reverse transcription was done using high-Capacity cDNA Reverse Transcription Kit (#4374966) and Real-time PCR (RT-PCR) using TaqMan™ Universal PCR Master Mix (#4369016), both from Applied Biosystems (Carlsbad, CA, USA). The primer sets used for BAT or hypothalamus samples are described in **Supplementary Table 2**. We used the data assist software from Applied Biosystems (CA, USA) to determine relative gene expression levels of *Ucp1* in BAT and *Agrp*, *Npy*, *Pomc*, and *Crh* in the hypothalamus. Gene expression was calculated through 2^-ΔCT^, in which ΔCT value is the difference between the threshold cycle (CT) value of the gene of interest and the CT value of the housekeeping gene.

### 2.11 Behavioral and emotional evaluation using open-field test

The OFT is the most used test in behavioral pharmacology to measure changes in locomotor activity, exploratory behavior, and emotionality, assessing the anxiety-like behavior of rodents (50–52). We performed the OFT based on the previous study (53). Briefly, Vgat-Cre; Clk2^loxP/loxP^, and Clk2^loxP/loxP^ mice at 12-14 weeks were placed individually in an open-field arena (50 cm x 48 cm x 50 cm; Insight, Brazil) with 6 bars, 16 infrared sensors, and luminosity (60 lx in the center) to explore the environment for 5 min. We determined the locomotor activity by tracking the traveled distance, total ambulatory activity, time of activity, average speed, and time spent in the center of the arena using the software Insight (USB, Ribeirão Preto, SP, Brazil). Furthermore, the time spent in the center and the number of fecal boluses left in the arena were used to determine emotional behavior (54, 55).

### 2.12 Spatial learning and memory evaluation using the Morris Water Maze test

The MWM is widely used to assess “normal” and pathological cognitive processes that influence learning and memory in rodents (56). MWM might be used to access spatial learning and memory in mice, providing an experimental approach to analyze the influence of genes, environmental factors, and their interactions on learning and memory (51). The test was performed in a black circular pool (170 cm in diameter) filled with water (22 °C). The circle delineating the pool was divided into four quadrants with four starting points, and four different distal cues represented each quadrant. An escape platform was placed into the water in one of the four quadrants. Mice were placed into the pool for 8 consecutive days, and data were recorded using a video camera fixed to the room’s ceiling and connected to a computer. On the first day, mice were allowed to swim individually for 60 s without the escape platform. From day 2 to day 7, mice followed the plan outlined and were allowed to swim until to climb onto the hidden platform. The time spent until the mouse reached the platform was recorded. Each trial finished when the animal climbed onto the hidden platform. On day 8, mice were tested for swimming for 120 s without an escape platform, and the time spent in the quadrant where the platform used to be, was recorded. In the end, the working memory/spatial learning was analyzed by evaluating each animal’s performance from day 1 to day 4, and the reference memory by evaluating the performance from day 4 to day 7 (57).

### 2.13 Statistical analysis

Results were displayed as the arithmetic mean ± standard error of the mean (SEM) and tested for normal distribution. To compare data from GABAergic neuron-specific CLK2 knockout mice (Vgat-Cre; Clk2^loxP/loxP^) and their controls (Clk2^loxP/loxP^) in both genders, we used two-tailed Student’s t-test, one-way or two-way analysis of variance (ANOVA) with Bonferroni post-test, as appropriate, throughout this study. All data were analyzed using GraphPad Prism (GraphPad Software, La Jolla, CA, USA), and the differences were considered statistically significant when P <0.05. The sample size (n) was adequate for the statistical tests used in the experimental conditions. All statistical tests and the sample size are indicated in figure captions.

## 3 Results

### 3.1 Deletion of CLK2 in GABAergic neurons increases adiposity in females but not in male mice

**Figure 1:**
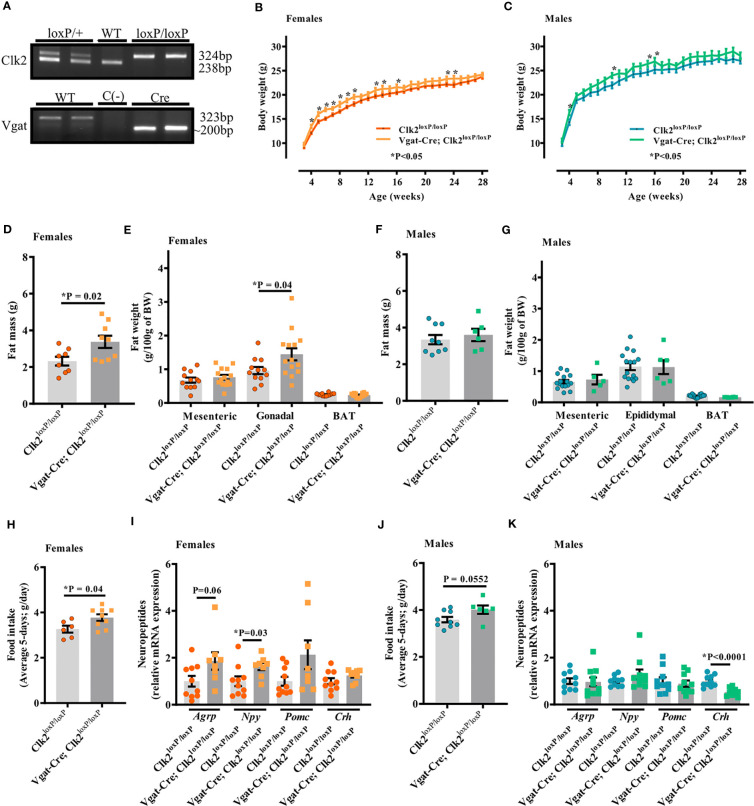
Deletion of CLK2 in GABAergic neurons leads to increased adiposity in female mice. **(A)** PCR analysis of mice tails genomic DNA. Clk2: the 238 bp and 324 bp bands correspond to the wild type (WT) or the floxed (loxP) alleles, respectively. Vgat: the 323 bp and ~200 bp bands correspond to the WT or the Vgat-Cre (Vgat^Cre^) alleles, respectively. **(B, C)** Body weight (g) throughout the study (females **(B)** n = 16 for Vgat-Cre; Clk2^loxP/loxP^, n = 12 for Clk2^loxP/loxP^; males **(C)** n = 6 for Vgat-Cre; Clk2^loxP/loxP^, n = 15 for Clk2^loxP/loxP^); **(D, F)** Total fat mass (g) at DEXA analysis day at a mean age of 23 weeks (females **(D)**: n = 9 for Vgat-Cre; Clk2^loxP/loxP^, n = 8 for Clk2^loxP/loxP^; males **(F)** n = 6 for Vgat-Cre; Clk2^loxP/loxP^, n = 9 for Clk2^loxP/loxP^); **(E, G)** Fat weight composition (g/100 g of BW), represented by mesenteric (MAT), gonadal/epidydimal (GAT/EAT) and brown (BAT) adipose tissue at 25-28 weeks of age (females **(E)** n = 14 for Vgat-Cre; Clk2^loxP/loxP^, n = 12 for Clk2^loxP/loxP^; males **(G)** n = 5-6 for Vgat-Cre; Clk2^loxP/loxP^, n = 16 for Clk2^loxP/loxP^); **(H, J)** Average daily food intake (average 5-days; g/day) at 16-18 weeks of age in both genders (females **(H)** n = 9 for Vgat-Cre; Clk2^loxP/loxP^, n = 6 for Clk2^loxP/loxP^; males **(J)** n = 6 for Vgat-Cre; Clk2^loxP/loxP^, n = 9 for Clk2^loxP/loxP^); **(I, K)** mRNA levels of neuropeptides (*Agrp* – *Agouti-related peptide*, *Crh* – a *corticotropin-releasing hormone*, *Npy* – neuropeptide Y, *Pomc* – proopiomelanocortin) determined by RT-PCR in hypothalamus of mice at 26-28 weeks of age (females **(I)**: n = 5 for Vgat-Cre; Clk2^loxP/loxP^, n = 5 for Clk2^loxP/loxP^; males **(K)** n = 5 for Vgat-Cre; Clk2^loxP/loxP^, n = 5 for Clk2^loxP/loxP^). Two-way ANOVA analyzed data from **(B)** and **(C)** with the Bonferroni post-test. Two-tailed unpaired Student’s t-test analyzed data from **(D–K)**. All bars and errors represent mean ± SEM. *P < 0.05 *versus* Clk2^loxP/loxP^.

As shown in **Figure 1A** (and **Supplementary Figure 1**), 324 bp from *Clk2* plus 200 bp products from *Vgat-Cre* have indicated GABAergic neuron-specific CLK2 knockout mice (Vgat-Cre; Clk2^loxP/loxP^), and a single 324 bp product indicating mice carrying the floxed allele from Clk2 (Clk2^loxP/loxP^) used as a control group.

We recorded the body weight weekly throughout 26-28 weeks of age. We observed a significant increase in body weight of Vgat-Cre; Clk2^loxP/loxP^ females between 4 and 10, and, at 13, 14, 16, 23, and 24 weeks of age compared with Clk2^loxP/loxP^ females (**Figure 1B**). In male mice, significant differences in body weight were observed at 4, 10, 15, and 16 weeks of age, with Vgat-Cre; Clk2^loxP/loxP^ males weighing more than the control mice (**Figure 1C**). Vgat-Cre; Clk2^loxP/loxP^ females exhibited a significant increase in total fat mass (**Figure 1D**) and similar values in lean mass (0.816 ± 0.014 g in Vgat-Cre; Clk2^loxP/loxP^, n = 9, and 0.846 ± 0.014 g in Clk2^loxP/loxP^, n = 8; P = 0.14) compared to Clk2^loxP/loxP^ females, both recorded by DEXA. The increase in fat mass was at least partially due to the significant enlargement in the gonadal adipose tissue but not dependent on mesenteric or BAT of Vgat-Cre; Clk2^loxP/loxP^ females compared to Clk2^loxP/loxP^ females (**Figure 1E**). For male mice, we did not observe any significant changes in the total fat mass recorded by DEXA (**Figure 1F**), nor lean mass (0.833 ± 0.010 g in Vgat-Cre; Clk2^loxP/loxP^, n = 6, and 0.841 ± 0.010 g in Clk2^loxP/loxP^, n = 9; P = 0.60), as well as in individual fat depots (mesenteric, epididymal and BAT adipose fat pads) (**Figure 1G**) between male mice genotypes.

To investigate the mechanism by which we found differences in the body weight between Vgat-Cre; Clk2^loxP/loxP^, and Clk2^loxP/loxP^ mice, we recorded food intake (g) for five consecutive days. We observed a significant increase in the average daily food intake (g) in Vgat-Cre; Clk2^loxP/loxP^ females compared to control females (**Figure 1H**). The average daily caloric intake was also high (P = 0.04) in the Vgat-Cre; Clk2^loxP/loxP^ females (12.79 ± 0.49 kcal/d; n=9) compared with control females (11.07 ± 0.52 kcal/d; n=6). Hyperphagia in Vgat-Cre; Clk2^loxP/loxP^ females could be associated with changes in orexigenic neuropeptides (**Figure 1I**), such as the significant increase of *Npy* and a tendency to increase (P=0.06) in *Agrp* mRNA levels (**Figure 1I**). We observed a trend toward an increase (P = 0.055) in daily food intake (g) of male Vgat-Cre; Clk2^loxP/loxP^ mice compared to control males (**Figure 1J**). The average daily caloric intake also showed a trend to be higher (P = 0.055) in Vgat-Cre; Clk2^loxP/loxP^ males (13.61 ± 0.602 kcal/d, n = 6) than in control males (12.17 ± 0.394 kcal/d, n = 9). The orexigenic neuropeptides, *Agrp*, and *Npy* mRNA levels were similar between male mice genotypes (**Figure 1K**). However, hypothalamic *Crh* gene expression was significantly lower in Vgat-Cre; Clk2^loxP/loxP^ males than in control males (**Figure 1K**). As CRH is a neuropeptide with anorexigenic function, leading to inhibition of hunger sensation, these data could be involved in the trend of increased food intake by Vgat-Cre; Clk2^loxP/loxP^ males in comparison to control males (**Figures 1J, K**).

### 3.2 Deletion of CLK2 in GABAergic neurons does not affect EE in females but increases in male mice during the dark cycle

**Figure 2:**
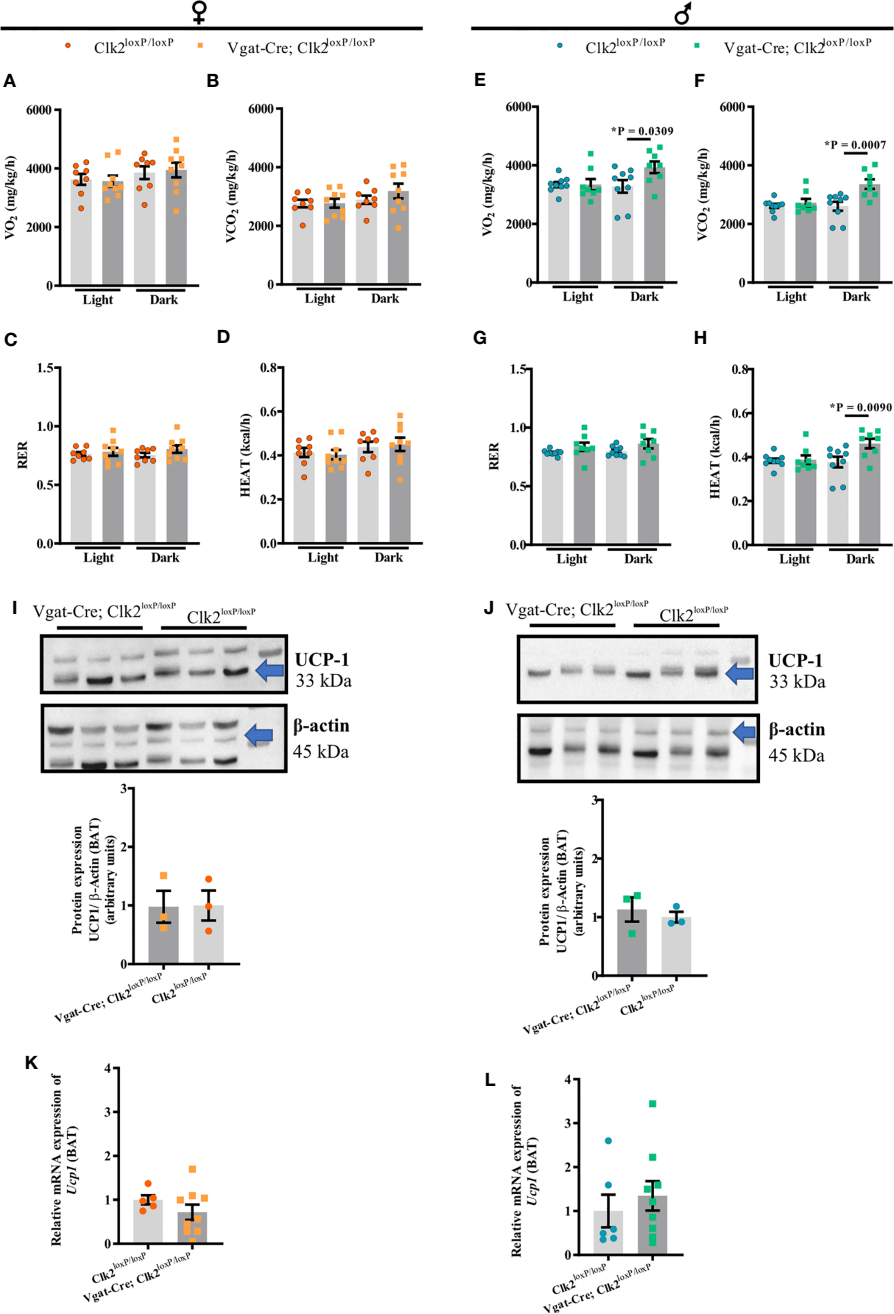
Deletion of CLK2 in GABAergic neurons does not affect female energy expenditure but increases in male mice during the dark cycle. Energy expenditure during light/dark cycle: **(A, E)** oxygen consumption (VO_2_), **(B, F)** carbon dioxide production (VCO_2_), **(C, G)** respiratory exchange ratio (RER) and, **(D, H)** heat production (HEAT) were measured for 24 h in both genders at 16-18 weeks of age (females **(A–D)** n = 9 for Vgat-Cre; Clk2^loxP/loxP^, n = 8 for Clk2^loxP/loxP^; males **(E–H)** n = 8 for Vgat-Cre; Clk2^loxP/loxP^, n = 9 for Clk2^loxP/loxP^); **(I, J)** protein expression of UCP-1 in BAT of overnight-fasted mice of both genders at 26-28 weeks of age (females **(I)**: n = 3 for Vgat-Cre; Clk2^loxP/loxP^, n = 3 for Clk2^loxP/loxP^; males **(J)** n = 3 for Vgat-Cre; Clk2^loxP/loxP^, n = 3 for Clk2^loxP/loxP^) represented by labeled band at 33 kDa (UCP-1) and a loading control at 45 kDa (β-Actin); **(K, L)** relative expression of *Ucp1* in BAT of mice of both genders at 26-28 weeks of age (females **(K)** n = 9 for Vgat-Cre; Clk2^loxP/loxP^, n = 5 for Clk2^loxP/loxP^; males **(L)** n = 9 for Vgat-Cre; Clk2^loxP/loxP^, n = 6 for Clk2^loxP/loxP^). All bars and errors represent mean ± SEM. Two-way ANOVA analyzed data from **(A–K)** following the Bonferroni post-test and data from **(I–L)** by two-tailed unpaired Student’s t-test. *P < 0.05 *versus* Clk2^loxP/loxP^.

To further investigate the mechanism by which we found differences in the body weight between Vgat-Cre; Clk2^loxP/loxP^, and Clk2^loxP/loxP^ mice, we recorded energy expenditure by indirect calorimetry in mice under 16-18 weeks of age. No significant VO2, VCO2, RER, and HEAT changes were found between female Vgat-Cre; Clk2^loxP/loxP^ mice and female control mice during the light and dark cycle (**Figures 2A–D**). Unlike the females, male Vgat-Cre; Clk2^loxP/loxP^ mice displayed elevated VO2, VCO2, and HEAT than control males during the dark cycle (**Figures 2E, F, H**). No changes in RER were seen between male mice genotypes (**Figures 2G**). Despite UCP-1 being an important mediator of thermogenesis in BAT, linked to basal and inducible energy expenditure [62-64], neither protein expression (females **Figure 2I** and males **Figure 2J** and **Supplementary Figure 2**) nor Ucp-1 mRNA (females **Figure 2K** and males **Figure 2L**) levels in BAT were different between genotypes in both genders.

### 3.3 Deletion of CLK2 in GABAergic neurons does not change fasting leptin and insulin levels in both genders

**Figure 3:**
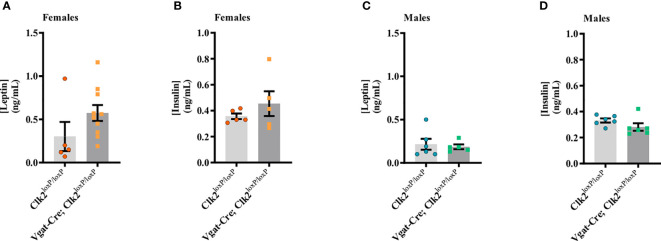
Deletion of CLK2 in GABAergic neurons does not change fasting leptin and insulin levels in both genders. **(A, C)** Fasting serum leptin (ng/mL) levels were measured by ELISA between 26-28 weeks of age (females **(A)** n = 10 for Vgat-Cre; Clk2^loxP/loxP^, n = 5 for Clk2^loxP/loxP^; males **(C)** n = 6 for Vgat-Cre; Clk2^loxP/loxP^, n = 5 for Clk2^loxP/loxP^); **(B, D)** Fasting serum insulin (ng/mL) levels were measured by ELISA between 26-28 weeks of age (females **(B)** n = 5 for Vgat-Cre; Clk2^loxP/loxP^, n = 5 for Clk2^loxP/loxP^; males **(D)** n = 6 for Vgat-Cre; Clk2^loxP/loxP^, n = 6 for Clk2^loxP/loxP^). Data were analyzed by two-tailed unpaired Student’s t-test. All bars and errors represent mean ± SEM.

There were no differences in fasting serum leptin levels (P = 0.1437; **Figure 3A**) or fasting serum insulin levels (P = 0.3486; **Figure 3B**) between the female genotypes. Among male mice genotypes, fasting serum leptin (P = 0.7037; **Figure 3C**) and fasting serum insulin (P = 0.4827; **Figure 3D**) levels were similar.

### 3.4 Deletion of CLK2 in GABAergic neurons leads to glucose intolerance but not insulin resistance in females

**Figure 4:**
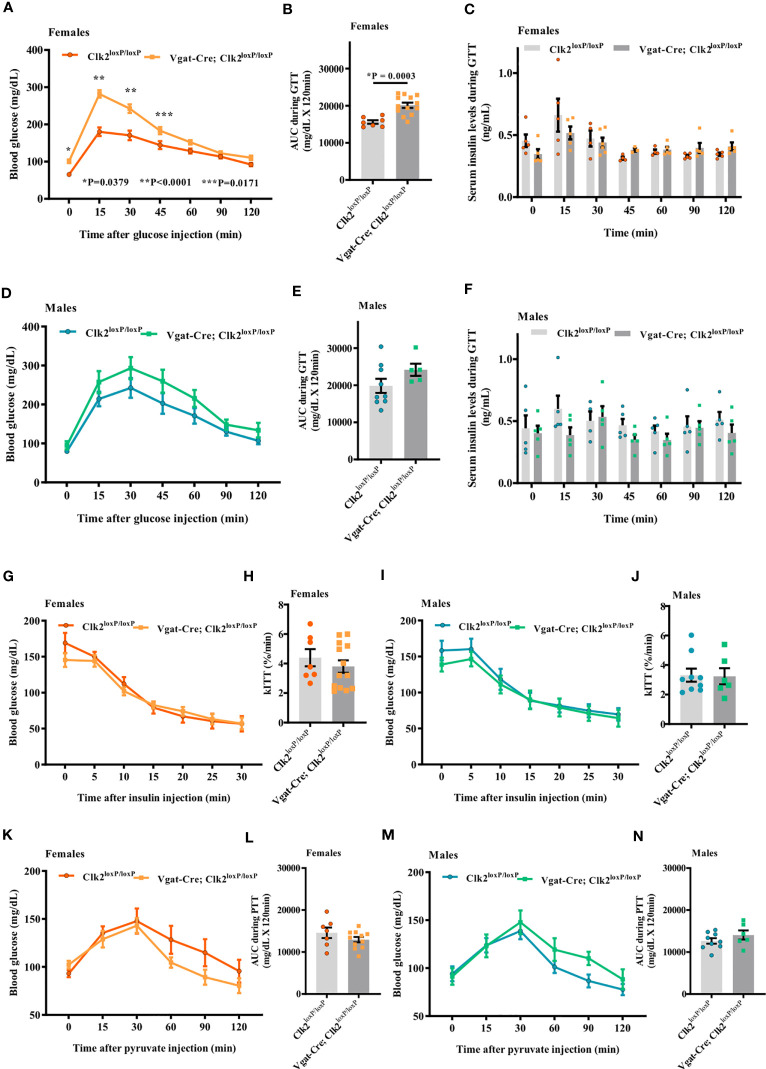
Deletion of CLK2 in GABAergic neurons leads to glucose intolerance but not insulin resistance in females. **(A, D)** Determination of blood glucose (mg/dL) during glucose tolerance test (GTT) in both genders of mice at 8 weeks of age (females **(A)** n = 13 for Vgat-Cre; Clk2^loxP/loxP^, n = 7 for Clk2^loxP/loxP^; males **(D)** n = 5 for Vgat-Cre; Clk2^loxP/loxP^, n = 9 for Clk2^loxP/loxP^); **(B, E)** Area under the curve (AUC) for glucose following GTT (mg/dL x 120 min); **(C, F)** Serum insulin levels (ng/mL) during GTT in mice at 8 weeks of age, determined by ELISA (females **(C)** n = 3-6 for Vgat-Cre; Clk2^loxP/loxP^, n = 4-5 for Clk2^loxP/loxP^; males **(F)** n = 5 for Vgat-Cre; Clk2^loxP/loxP^, n = 4-5 for Clk2^loxP/loxP^). **(G, I)** Blood glucose (mg/dL) during insulin tolerance test (ITT) in both genders of mice at 9 weeks of age (females **(G)** n = 13 for Vgat-Cre; Clk2^loxP/loxP^, n = 7 for Clk2^loxP/loxP^; males **(I)** n = 6 for Vgat-Cre; Clk2^loxP/loxP^, n = 9 for Clk2^loxP/loxP^); **(H, J)** Glucose decay constant during ITT (k ITT) (%/min); **(K, M)** Blood glucose (mg/dL) during pyruvate tolerance test (PTT) in both genders of mice at 10 weeks of age (females **(K)** n = 11 for Vgat-Cre; Clk2^loxP/loxP^, n = 7 for Clk2^loxP/loxP^; males **(M)** n = 6 for Vgat-Cre; Clk2^loxP/loxP^, n = 9 for Clk2^loxP/loxP^); **(L, N)** Area under the curve (AUC) for glucose following PTT (mg/dL x 120 min). All bars and errors represent mean ± SEM. Data from **(A, D, G, I, K, M)** were analyzed by two-way ANOVA with Bonferroni post-test. *P = 0.0379, **P <0.0001, ***P = 0.0171 *versus* Clk2^loxP/loxP^. Data from **(B, C, E, F, H, J, L, N)** were analyzed by two-tailed unpaired Student’s t-test. *P <0.05 *versus* Clk2^loxP/loxP^. A single dose of insulin (1.0 IU/kg of body weight; Human recombinant insulin, Eli Lilly) or sodium pyruvate (1.5 g/kg of BW; ReagentPlus^®^, Sigma-Aldrich) were IP administered in each animal after fasting blood glucose measurements.

Baseline fasting blood glucose levels were significantly higher (P <0.0001) in Vgat-Cre; Clk2^loxP/loxP^ females (101.0 ± 4.76 mg/dL, n = 13) than in control females (65.43 ± 3.82 mg/dL, n = 7) (**Figure 4A**). Blood glucose at 15, 30, and 45 min after IP glucose administration were significantly higher in Vgat-Cre; Clk2^loxP/loxP^ females than in control females (**Figure 4A**). AUC was higher in the Vgat-Cre; Clk2^loxP/loxP^ females than in controls, suggesting glucose intolerance in the female Vgat-Cre; Clk2^loxP/loxP^ mice (**Figure 4B**). Additionally, we determined the serum insulin levels using blood samples collected during GTT, and no changes in serum insulin concentrations were observed in female groups (**Figure 4C**).

Although blood glucose levels in male Vgat-Cre; Clk2^loxP/loxP^ mice were higher than in control mice throughout the GTT period, these values did not reach statistical significance between groups of males (**Figure 4D**). AUC was not different between male mice genotypes during GTT (**Figure 4E**). We also determined serum insulin levels using blood samples collected during GTT, and no changes in serum insulin concentrations were observed in any of the male groups (**Figure 4F**).

At 9 weeks of age, we recorded blood glucose in response to insulin administration (1.0 IU/kg of BW) as an indicator of peripheral insulin sensitivity. Blood glucose before and after IP administration (0, 5, 10, 15, 20, 25, and 30 minutes) of insulin was not different between female groups (**Figure 4G**). The glucose decay constant rate (kITT) during ITT was similar between female genotypes (**Figure 4H**). Similarly, we did not observe any differences between male genotypes regarding blood glucose before and after IP insulin administration (**Figure 4I**) nor kITT during ITT in male mice from both genotypes (**Figure 4J**).

At 10 weeks of age, we performed PTT to investigate, at least indirectly, the hepatic glucose production in mice with deletion of CLK2 in GABAergic neurons in both genders. Blood glucose levels before and after IP pyruvate administration (0, 15, 30, 60, 90, and 120 minutes) were not different (females **Figure 4K** and males **Figure 4M**), as well as AUC of blood glucose during PTT, between distinct genotypes from both genders (females **Figure 4L** and males **Figure 4N**).

Together, these data suggest that CLK2 in GABAergic neurons may regulate glucose tolerance in a sex-dependent manner. However, CLK2 may not regulate insulin sensitivity and hepatic glucose production.

### 3.5 The deletion of CLK2 in GABAergic neurons in female mice might affect the hypothalamic insulin action and signaling

To determine whether the deletion of CLK2 in GABAergic neurons affects insulin action on feeding behavior, we measured food intake during 4, 8, 12, and 24h in response to ICV insulin injection in Clk2^loxP/loxP^ and Vgat-Cre; Clk2^loxP/loxP^ mice from both genders.

**Figure 5:**
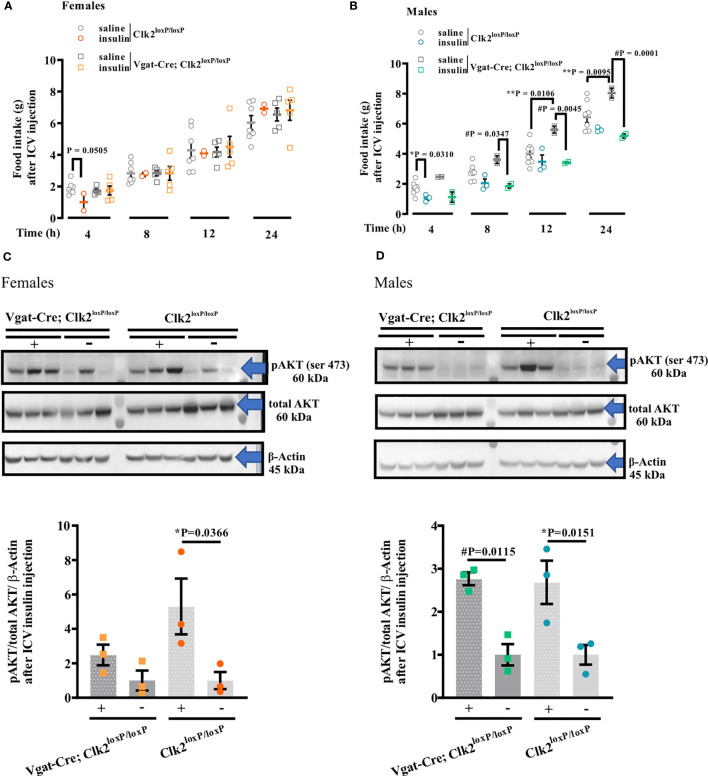
Deleting CLK2 in GABAergic neurons in female and male mice may affects hypothalamic insulin action and signaling. **(A, B)** food intake after 4, 8, 12, and 24 h of a single ICV injection of insulin (0.2 μg of Human recombinant insulin, Eli Lilly) in comparison to saline (0.9% NaCl). **(C, D)** AKT phosphorylation (pAKT) in the hypothalamus analyzed by Western Blotting following 15 min of ICV insulin (0.2 μg) or saline administration. All bars and errors represent mean ± SEM. Data from A and B were analyzed by two-tailed Student’s t-test, and from C and D were analyzed by one-way ANOVA with Bonferroni post-test. *P <0.05: Clk2^loxP/loxP^ (insulin) *versus* Clk2^loxP/loxP^ (saline); #P <0.05: Vgat-Cre; Clk2^loxP/loxP^ (insulin) versus Vgat-Cre; Clk2^loxP/loxP^ (saline); **P <0.05: Clk2^loxP/loxP^ (saline) *versus* Vgat-Cre; Clk2^loxP/loxP^ (saline). The experiments were performed in females **(A, C)** or males **(B, D)** at 26-28 weeks of age after overnight fasting (8:00 PM to 9:00 AM). pAKT Ser473 (#9271s – 60 kDa), total AKT (pan; 11E7, #4685S – 60 kDa), and β-Actin (#4967 – 45 kDa) antibodies from Cell Signaling Technology (Boston, MA, USA). N for females **(A)**: Clk2^loxP/loxP^ (saline): 7 and (insulin): 2. Vgat-Cre; Clk2^loxP/loxP^ (saline): 5 and (insulin): 5. N for males **(B)** Clk2^loxP/loxP^ (saline): 8 and (insulin): 3. Vgat-Cre; Clk2^loxP/loxP^ (saline): 2 and (insulin): 2. N for females **(C)** and males **(D)** 3 for each group. '#' represents significant differences between males Vgat-Cre; Clk2^loxP/loxP^, which received saline or insulin.

In females, ICV insulin injection (0.2 μg/μL) decreased food intake after 4 h compared to saline in the control mice (**Figure 5A**). We did not observe any further differences in food intake between females in response to insulin compared to saline in both genotypes at 8, 12, and 24 h (**Figure 5A**). In males, we observed a decrease (P = 0.0310) in food intake in response to insulin in the control group (Clk2^loxP/loxP^) 4 h after ICV insulin injection compared to saline-injected control mice. However, we did not observe differences (P>0.05) in food intake in response to insulin in the control group (Clk2^loxP/loxP^) at 8, 12, and 24 h after ICV insulin injection compared to saline-injected control mice (**Figure 5B**). In contrast, male Vgat-Cre; Clk2^loxP/loxP^ mice significantly reduced their food intake in response to insulin at 8 h (P = 0.0347), 12 h (P = 0.0106), and 24 h (P = 0.0001) after ICV insulin injection compared to saline-injected Vgat-Cre; Clk2^loxP/loxP^ mice (**Figure 5B**).

In females, accordingly, to the physiological response to ICV insulin, we observed an increase in Akt phosphorylation in the hypothalamus of female control mice compared to mice injected with saline (**Figure 5C**, **Supplementary Figure 3**). This response was abolished in Vgat-Cre; Clk2^loxP/loxP^ mice (**Figure 5C**, **Supplementary Figure 3**).

In male mice, we correspondingly observed an increase in Akt phosphorylation in the hypothalamus of male control mice compared to mice injected with saline (**Figure 5D**, **Supplementary Figure 3**). However, in male Vgat-Cre; Clk2^loxP/loxP^ mice, we observed a similar increase in Akt phosphorylation in the hypothalamus compared to Vgat-Cre; Clk2^loxP/loxP^ injected with saline (**Figure 5D**, **Supplementary Figure 3**).

On the one hand, the deletion of CLK2 in GABAergic neurons does not seem to compromise hypothalamic insulin action in male mice. On the other hand, hypothalamic insulin signaling, and consequently its action, appears to be compromised in females lacking CLK2 in GABAergic neurons.

Together, these data suggest that CLK2 may have a role in the insulin action and signaling in GABAergic neurons from the hypothalamus of females but not males.

### 3.6 Deletion of CLK2 in GABAergic neurons leads to less anxiety-like behavior in female mice

**Figure 6:**
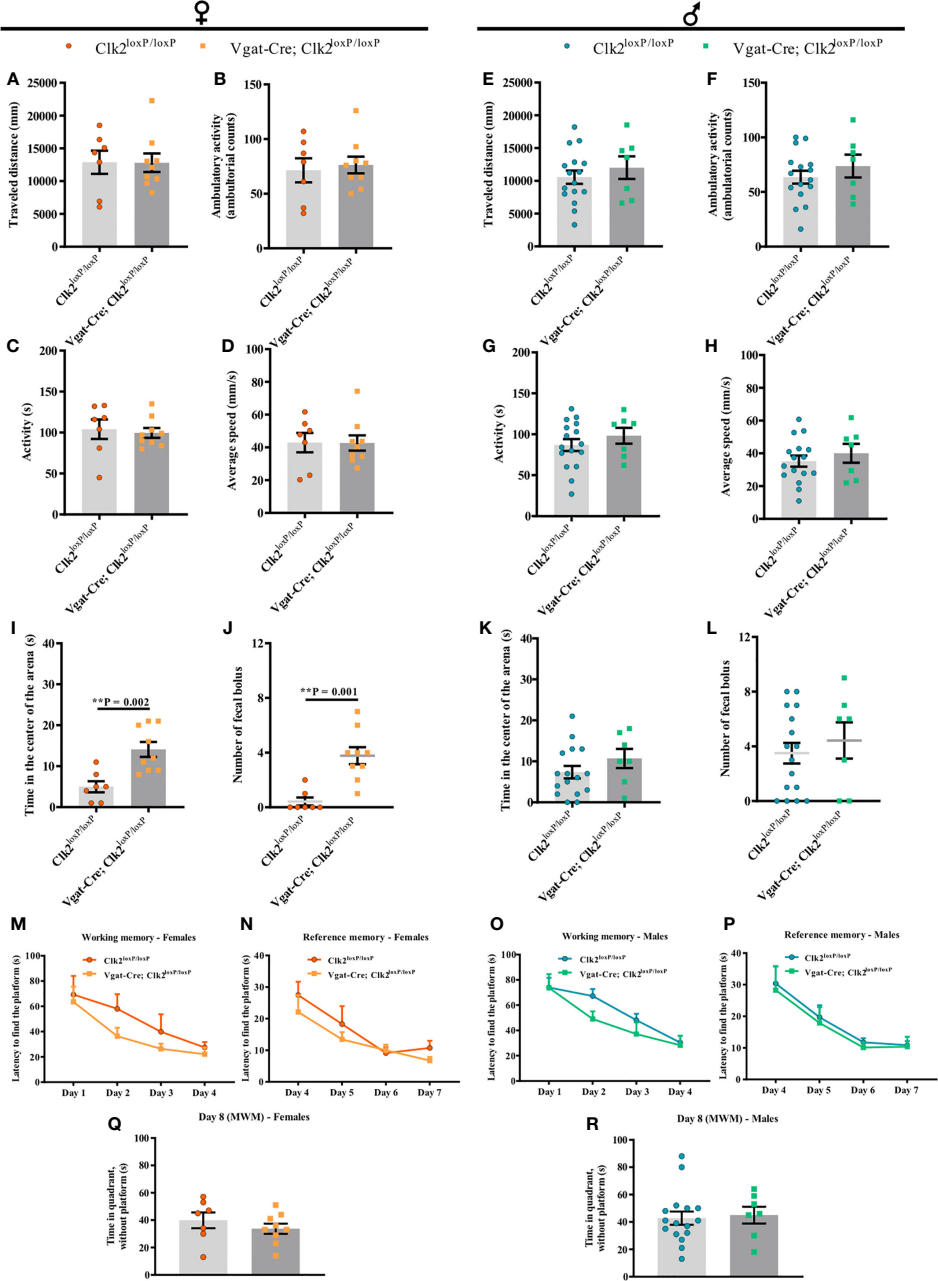
The lack of CLK2 in GABAergic neurons leads to less anxiety-like behavior in female mice without affecting learning and memory abilities. **(A, E)** traveled distance (mm); **(B, F)** ambulatory activity (ambulatory counts); **(C, G)** activity (s); **(D)** and **(H)** average speed (mm/s); **(I, K)** time (s) spent in the center of the arena by mice; **(J, L)** number of fecal boluses left in the arena and recorded during Open-field test (OFT) in female and male mice, respectively; **(M, O)** spatial learning/working memory analyzed by latency (s) to find the platform for 4 daily trials solved from day 1 to day 4 in female **(M)** and male **(O)** mice; **(N, P)** reference memory analyzed by latency (s) from day 4 to day 7 in females **(N)** and males **(P)**; **(Q, R)** time (s) spent by females **(Q)** and males **(R)** in the quadrant without platform (day 8), where the platform used to be on the last 4 days. All experiments were recorded in control (Clk2^loxP/loxP^) and knockout (Vgat-Cre; Clk2^loxP/loxP^) mice between 12-14 weeks of age. Data from **(A-L)** were recorded during OFT (**A–L**; females **(A–D, I, J)**: n = 9 for Vgat-Cre; Clk2^loxP/loxP^, n = 7 for Clk2^loxP/loxP^; males **(E–H, K, L)**: n = 7 for Vgat-Cre; Clk2^loxP/loxP^, n = 16 for Clk2^loxP/loxP^). M-R data were recorded during the Morris Water Maze (MWM) test (**M–R**; females **(M, N, Q)**: n = 9 for Vgat-Cre; Clk2^loxP/loxP^, n = 7 for Clk2^loxP/loxP^; males **(O, P, R)**: n = 7 for Vgat-Cre; Clk2^loxP/loxP^, n = 16 for Clk2^loxP/loxP^). All bars and errors represent mean ± SEM. **(A- L, Q, R)** data were analyzed by a two-tailed Student’s t-test, except data from **(J)**, which was analyzed by a two-tailed Mann-Whitney test. **(M-P)** data were analyzed by repeated measures two-way ANOVA with Bonferroni post-test. **P <0.01 *versus* Clk2^loxP/loxP^.

To measure locomotor activity and emotionality and assess the anxiety-like behavior of mice lacking CLK2 in GABAergic neurons at young adulthood age, we performed the OFT. Mice were placed individually in an open-field arena to explore the environment for 5 min. After analyzing the locomotor activity, we observed that traveled distance, ambulatory activity, time of activity, and average speed were similar in both genotypes of female mice (**Figures 6A–D**).

In males, we observed similar results as females, with no differences in the traveled distance, ambulatory activity, time of activity, and average speed in both genotypes of male mice (**Figures 6E–H**).

To investigate anxiety-like behavior more specifically, we analyzed the time spent in the center of the arena during the OFT. Additionally, we measured the number of fecal boluses left on the arena during the OFT to deduce the emotional behavior of mice (58, 59).

In females, we observed that Vgat-Cre; Clk2^loxP/loxP^ mice significantly spent more time in the center of the arena than control females (**Figure 6I**). Female Vgat-Cre; Clk2^loxP/loxP^ displayed a significant increase in fecal bolus number left on the arena compared to control mice (**Figure 6J**).

In contrast, in male mice, we did not observe differences in the time spent in the center of the arena between both genotypes (**Figure 6K**). Likewise, there were no differences in the number of fecal boluses left on the arena between male mice genotypes (**Figure 6L**).

Our data suggest that the absence of CLK2 in GABAergic neurons may contribute to decreasing anxiety-like behavior and alter the emotional behavior only in females.

### 3.7 Deletion of CLK2 in GABAergic neurons does not affect learning and memory abilities

We analyzed whether the deletion of CLK2 in GABAergic neurons impacts spatial learning and memory abilities by testing spatial navigation in the MWM test. Mice learn the location of a hidden platform using distal cues. Mice lacking CLK2 in GABAergic neurons found the hidden platform at a similar rate, showing that mice have similar working and reference memory in both genders compared with their control mice (females **Figures 6M, N** and males **Figures 6O, P**). On day 8, all mice spent similar time in the quadrant where the platform was on the last 4 days, reinforcing similar hippocampal function (females **Figure 6Q** and males **Figure 6R**) among mice, independent of the genotype or gender.

## 4 Discussion

Our findings demonstrated that deletion of CLK2 in GABAergic neurons increases adiposity, likely owing to hyperphagia in females. Vgat-Cre; Clk2^loxP/loxP^ females are systemic glucose intolerant and traits toward decrease in hypothalamic response to insulin. Male Vgat-Cre; Clk2^loxP/loxP^ mice slightly increased body weight but similar fat mass to male control mice. Surprisingly, male Vgat-Cre; Clk2^loxP/loxP^ mice showed a significant increase in energy expenditure, which might account for keeping adiposity equivalent to the control mice despite a tendency (P = 0.0552) to increase food intake. Unlike females, male Vgat-Cre; Clk2^loxP/loxP^ mice have normal glucose tolerance. Vgat-Cre; Clk2^loxP/loxP^ mice had no alteration in cognition or memory functions in both genders. Interestingly, only females (not males) GABAergic neuron-specific CLK2 deficient mice exhibited anxiety-like behavior changes compared to their gender control.

Mice lacking CLK2 in the liver (30, 31, 33) or adipose tissue (34), or CLK2 knockdown in the hypothalamus (32) have been explored to elucidate the role of CLK2 in energy homeostasis. Our previous study confirmed that CLK2 was expressed in neurons, not in glial cells of the hypothalamus. The knockdown or pharmacological inhibition of CLK2 in the hypothalamus increased body weight and fat mass (32). Unfortunately, in our previous work, we checked only the male, not the female phenotype. In the present study, the effect of deletion of CLK2 in GABAergic neurons was pronounced in females, not males.

Similar to our result, female mice exhibiting insulin receptor deletion in GABAergic neurons (VgatIRKO) presented a significant increase in total body weight and fat mass (39). Because CLK2 participates in the insulin signaling pathway in several tissues (30–32, 34), the body weight regulation similarity among these female mice models was not surprising.

Our previous report showed that hypothalamic CLK2 participated in the insulin signaling cascade (32). Also, the anorexigenic effect of insulin was partially blunted by CLK2 inhibition (32). Here we observed a decreased response to ICV insulin injection in knockout females. Together, these data reinforced the CLK2 involvement in the insulin pathway regarding energy homeostasis control.

We detected increased *Npy* and *Agrp* expression in the hypothalamus of female knockout mice. This result might imply that both orexigenic neuropeptides are involved in the increased food intake of female knockout mice. AgRP/NPY neurons are predominantly GABAergic (22, 58). Recently, another GABAergic neuronal population, prepronociceptin-expressing neurons (PNOC), was described in the ARC. This new population of neurons differs from AgRP and POMC and promotes food intake, especially palatable food (59). Not only in the ARC but PNOC-expressing neurons in the central amygdala likewise increase food intake, in addition to rewarding (60). Our previous study observed a high expression of CLK2 in the amygdala, especially the CeA (32). Due to GABAergic neurons being broadly expressed, CLK2 deletion from these neurons may have affected regions other than ARC that are also involved in the feeding control.

The mechanisms behind feeding regulation may differ in both genders of Vgat-Cre; Clk2^loxP/loxP^ mice. In male knockout mice, we observed only a mild increased (P = 0.0552) food intake associated with a reduced expression of *Crh* in the hypothalamus. Since hypothalamic CRH decreases feeding (24, 25), we speculated that lower CRH levels in the hypothalamus might be behind the mild increase in the feeding of male knockout mice.

Contrary to females, male knockout mice did not display increased adiposity. This data recapitulates the metabolic phenotype of males VgatIRKO (39) and IR neuron-specific disruption (NIRKO) (61). Together, these data support the critical role of insulin signaling in GABAergic neurons to keep the energy balance.

Unlike females, males Vgat-Cre; Clk2^loxP/loxP^ had increased energy expenditure, possibly contributing to maintaining stable adiposity. POMC neurons are closely related to increased energy expenditure, while AgRP neurons are closely related to feeding behavior (62). POMC neurons are mainly glutamatergic (63) and, to a lesser extent, GABAergic (63). Therefore, it is unsurprising that we did not observe differences in *Pomc* levels in the hypothalamus in the knockouts compared to controls in both genders. These data suggest that POMC neurons in the hypothalamus may not be involved in determining energy expenditure in males. In females, this result was supported by similar UCP-1 expression (protein and gene expressions) in BAT.

Nevertheless, the higher energy expenditure in males was not related to the UCP-1 expression in BAT. Other cycles of energy expenditure in adipocytes and muscle (creatine and calcium cycle, known as the non-ATP-producing substrate cycle) can complement or replace UCP1-mediated thermogenesis (64–66). Alternatively, increased sympathetic nervous system activity may drive the high energy expenditure in males may be driven by increased sympathetic nervous system activity since BAT has marked sympathetic innervation (65, 67). Additional studies deciphering the mechanisms underlying the changes in energy expenditure in males lacking CLK2 in GABAergic neurons should be explored in the future.

Despite females Vgat-Cre; Clk2^loxP/loxP^ mice displaying increased adiposity, we did not find changes in peripheral insulin sensitivity. Insulin resistance usually accompanies the increase in adiposity (68). The female knockouts developed glucose intolerance despite normal insulin sensitivity, unlike VgatIRKO mice, which did not show glucose intolerance (39). The glucose intolerance in females Vgat-Cre; Clk2^loxP/loxP^ was not due to reduced glucose-induced insulin secretion since there were no differences in insulin concentrations during GTT. We speculate that glucose intolerance in the females may be partially due to impaired hypothalamic insulin signaling caused by the deletion of CLK2 in GABAergic neurons. The speculation also accounts for the male knockout result, which had normal glucose tolerance and hypothalamic insulin signaling. Since females Vgat-Cre; Clk2^loxP/loxP^ exhibited glucose intolerance without changes in the pyruvate response, it is suggested that the mechanism behind glucose intolerance differs from another proposed earlier (30–32). Hence further studies will be needed to unravel glucose uptake by other tissues.

The GABAergic neurons have been associated with behavioral and cognitive functions that regulate learning, vigilance, memory, anxiety, locomotion, feeding, and reward. Among the aspects of behavior studied in the present study (spatial learning and memory capacity, locomotion, and anxiety), the deletion of CLK2 in GABAergic neurons has only impacted anxiety-like behavior in the female (Vgat-Cre; Clk2^loxP/loxP^) mice group. During the OFT, females Vgat-Cre; Clk2^loxP/loxP^ spent more time in the center of the arena than control (Clk2^loxP/loxP^) females. Spending more time in the center of the arena or less time along the apparatus’s walls indicates less anxiety-like behavior (69, 70). Nevertheless, females Vgat-Cre; Clk2^loxP/loxP^ left more fecal boluses on the arena after OFT. Even though more fecal boluses suggest more anxiety (54, 55, 71), the knockout females ate more than the controls, which might justify an increased fecal bolus on the arena. Therefore, the higher fecal boluses left might be due to elevated gastric content or gut mobility and not a measure of anxiety.

When arguing about behavior, it is crucial to remember that food intake and emotion might be related components; the hypothalamus does not perform isolated functions. The hypothalamus controls ingestion through its connections with other brain areas. Furthermore, the hypothalamus responds according to the peripheral organs’ signals through autonomous and endocrine flow. However, emotional, cognitive, and executive support for feeding behavior is regulated by an interactive network involving the cortico-limbic system (including the amygdala), the hypothalamus, and the brainstem (72–75). In our previous study, we observed a high expression of CLK2 in the amygdala, especially the CeA, which is enriched with GABAergic neurons (32). The global deletion of CLK2 from GABAergic neurons affects several brain motivational areas, which might influence feeding.

Discrepancies in anxiety-like behavior found in females but not males suggest a potential sex dimorphism under the participation of CLK2 in behavioral regulation. It is known that males and females have differences in neuroanatomical and chemical signals, which might contribute to the gender distinctions we observed in behavioral responses (76–79). Additionally, sex hormones in both genders may affect many neural and behavioral functions (80, 81) and, particularly, might contribute to changing GABAergic circuit functions (82).

The limitation of this study is that GABAergic neurons are expressed throughout the brain and involve multiple kinds of neurons. Therefore, the regions and the identity of GABAergic neurons involved in our mice’s metabolic and behavioral phenotype are unknown. A future study will investigate this issue.

In conclusion, this study allows, for the first time, to characterize the metabolic phenotype of mice lacking CLK2 in GABAergic neurons, indicating GABAergic neuron-specific CLK2 deletion plays a critical role in keeping the energy homeostasis in female mice but not males. In female mice, the CLK2 in GABAergic neurons is required to maintain body weight, adiposity, and food intake. Our findings demonstrate how deletion of CLK2 in GABAergic neurons impairs these parameters, which could be sustained, at least in part, due to hypothalamic insulin resistance and upregulation of hypothalamic Npy and Agrp expression. Vgat-Cre; Clk2^loxP/loxP^ females were glucose intolerant, which appears to be independent of global insulin sensitivity and pyruvate response. In addition, loss of CLK2 in GABAergic neurons changes anxiety-like behavior only in female mice without compromising cognition or memory function. Thus, the current study suggests that CLK2 is a promising target in treating obesity associated with psychological disorders, at least in females.

## Data availability statement

The raw data supporting the conclusions of this article will be made available by the authors, without undue reservation.

## Ethics statement

The animal study was reviewed and approved by the ethics committee for Animal Experimentation at the University of Campinas (UNICAMP), São Paulo, Brazil, approved the experimental protocol used in this study (CEUA 5103-1/2018, CEUA 5102-1/2018 and CIBio 23/2018).

## Author contributions

SN and PP designed and planned all the experiments. SN maintained colonies of Clk2 ^loxP/loxP^ and Vgat-Cre; Clk2 ^loxP/loxP^ mice used during this study performed all the experiments, analyzed data, and wrote the manuscript. HA and DG performed animal care assistance and technical support of mice blood collection during GTT. HA performed assistance in food intake records, collection of sample tissues at final experiments, RNA extractions from BAT and WB experiments. DG performed leptin and insulin levels determinations. NT performed technical assistance using DEXA. PB provided equipment support and technical assistance for behavioral tests. M-CK generated Vgat-Cre and Clk2 floxed mice. MS provided material, equipment, and financial support. Y-BK reviewed the manuscript and provided the founder’s mice. PP directed the study, provided financial support, reviewed data analysis, and wrote the manuscript. All authors contributed to the article and approved the submitted version.
