# Supplementary material for: CLK2 in GABAergic neurons is critical in regulating energy balance and anxiety-like behavior in a gender-specific fashion
Source: Front Endocrinol (Lausanne). 2023 Aug 10;14:1172835. doi: 10.3389/fendo.2023.1172835 (PMC10449579; doi:10.3389/fendo.2023.1172835)
Supplement: Supplementary file 1 [file Table_1.docx]

**Supplementary Table 1.** Primer sets used for genotyping.

|  | *Primer Forward* (5’ – 3’) | *Primer Reverse* (5’ – 3’) |
| --- | --- | --- |
| Clk2 | GAGAATATGGGAGTCCTGGAACTGG | GAAGAGGTGGCAGGTGTTATCTCTGA |
| Vgat-IRES-Cre | CTTCGTCATCGGCGGCATCTG | CAGGGCGATGTGGAATAGAAA |
|  |  | CCAAAAGACGGCAATATGGT |
| Cre 349 | CCCGCAGAACCTGAAGATGT | TGATCCTGGCAATTTCGGCT |

Primers for Clk2 and Vgat-IRES-Cre were obtained from Integrated DNA Technologies (IDT^TM^) and for generic Cre 349 from Exxtend®, Brazil. For Vgat-IRES-Cre, three primers were used in two pairs combination according to the Jackson Laboratory’s instructions.
