# Supplementary material for: CLK2 in GABAergic neurons is critical in regulating energy balance and anxiety-like behavior in a gender-specific fashion
Source: Front Endocrinol (Lausanne). 2023 Aug 10;14:1172835. doi: 10.3389/fendo.2023.1172835 (PMC10449579; doi:10.3389/fendo.2023.1172835)
Supplement: Supplementary file 2 [file Table_2.docx]

**Supplementary Table 2.** Primer sets used for brown adipose tissue (BAT) or hypothalamus samples.

| Gene | *Primer Forward* (5’ – 3’) | *Primer Reverse* (5’ – 3’) |
| --- | --- | --- |
| *Agrp* | CTTTGGCGGAGGTGCTAGAT | AGGACTCGTGCAGCCTTACAC |
| *B2m* | ATGGAGCTCTGAATCATCTGG | AGAAGATGGTGTGCTCATTGC |
| *Crh* | AAAGCAGATGGGAGTCATCCA | GCCACCCCTCAAGAATGAATT |
| *Gapdh* | GGAGTCCACTGGCGTCTTCAC | GAGGCATTGCTGATGATCTTGAGG |
| *Npy* | CCCCAGAACAAGGCTTGAAG | TTGGAAAAGTCGGGAGAACAA |
| *Pomc* | TGGGCGAGCTGATGACCT | GCCGACTGTGAAATCTGAAAGG |
| *Ucp1* | GGCATTCAGAGGCAAATCAGCT | CAATGAACACTGCCACACCTC |

*Genes of interest: Agrp* – *Agouti-related peptide*, *Crh* – *corticotropin-releasing hormone*, *Npy* – neuropeptide Y, *Pomc* – proopiomelanocortin, *Ucp1* – *uncoupling protein 1*. Housekeeping genes: *B2m* – beta-2-microglobulin, *Gapdh* – *glyceraldehyde 3-phosphate dehydrogenase.*
