# Supplementary figures and images for: CLK2 in GABAergic neurons is critical in regulating energy balance and anxiety-like behavior in a gender-specific fashion
Source: Front Endocrinol (Lausanne). 2023 Aug 10;14:1172835. doi: 10.3389/fendo.2023.1172835 (PMC10449579; doi:10.3389/fendo.2023.1172835)

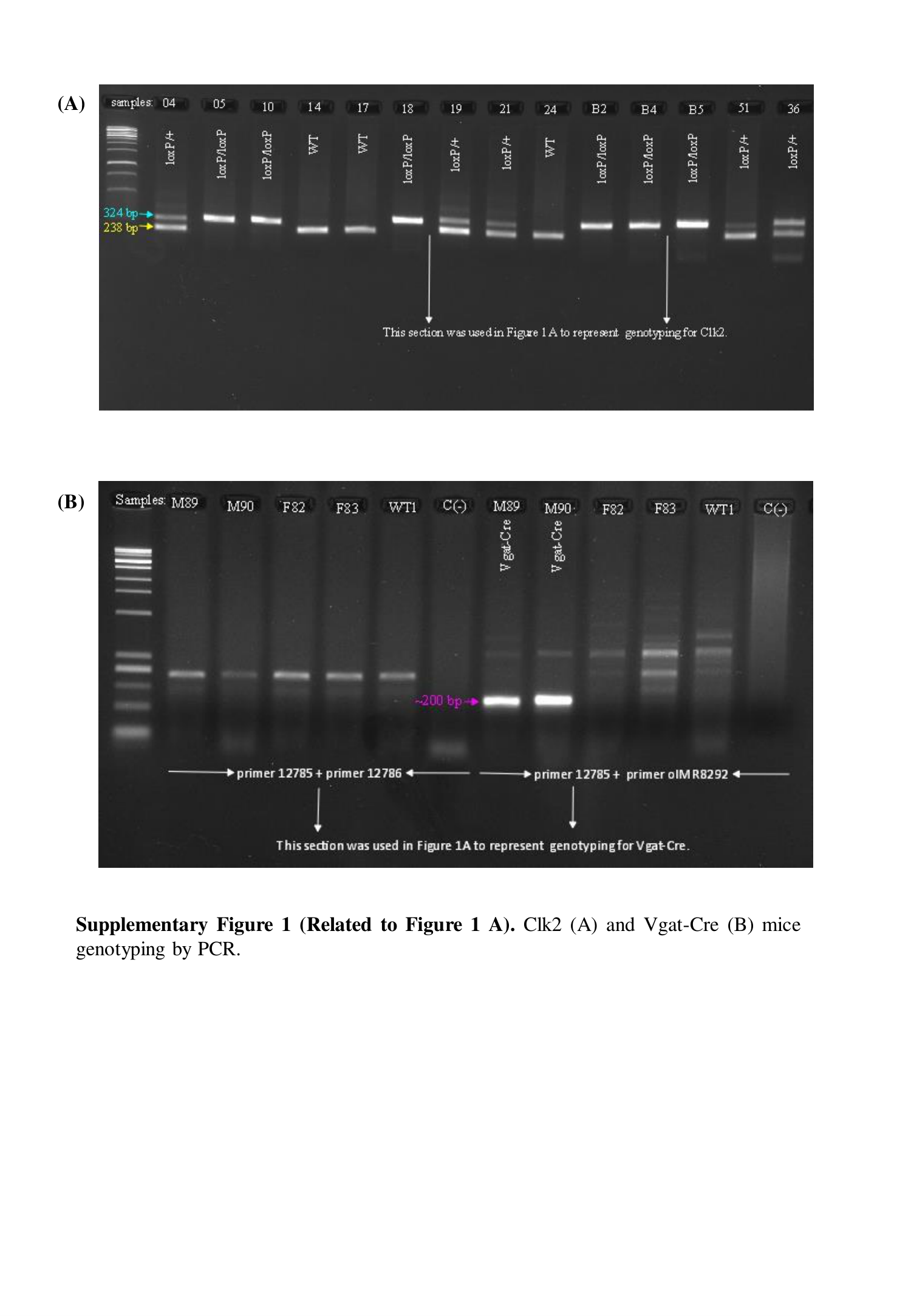

Supplement: Supplementary file 3 [file Image_1.tiff]

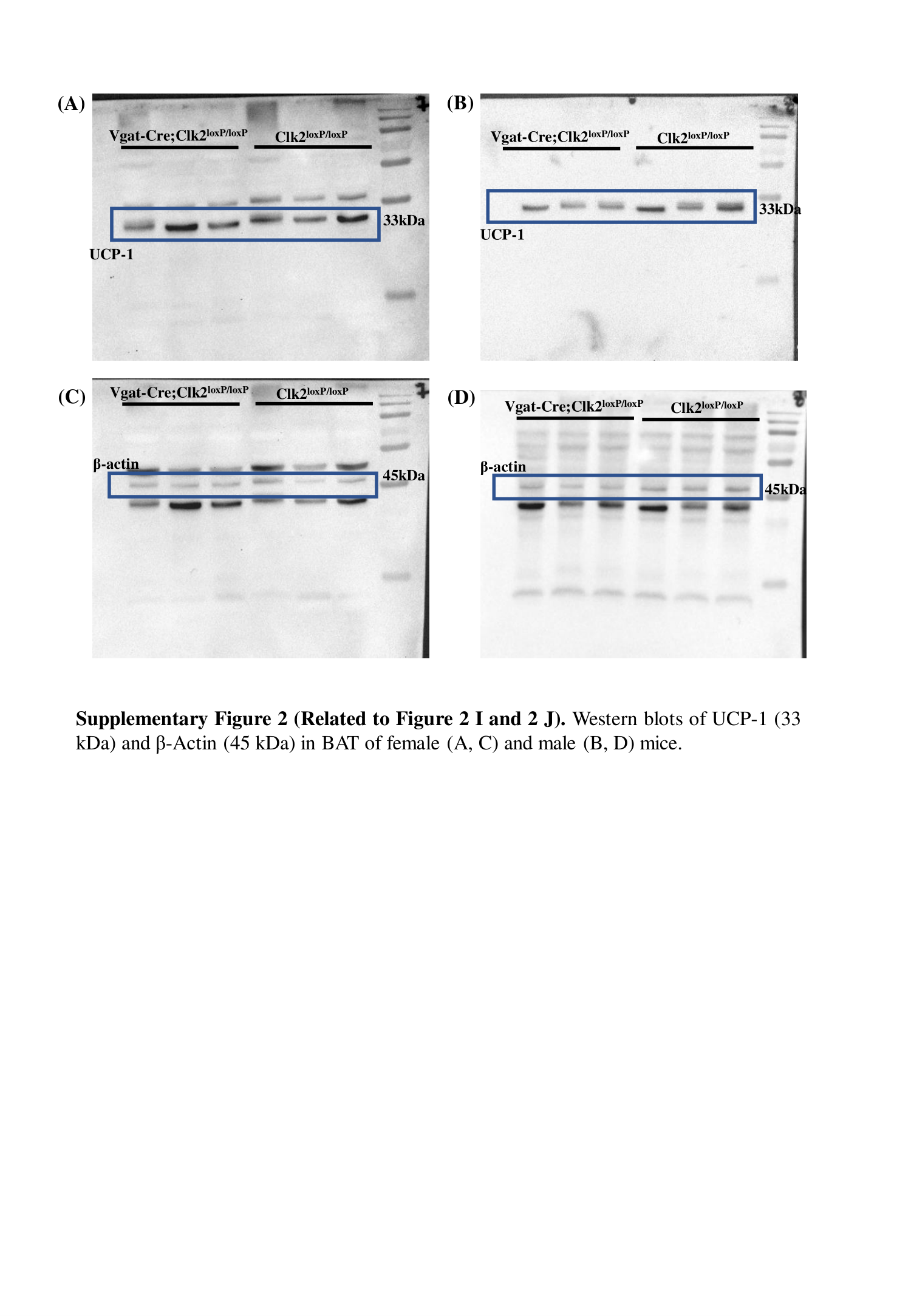

Supplement: Supplementary file 4 [file Image_2.tiff]

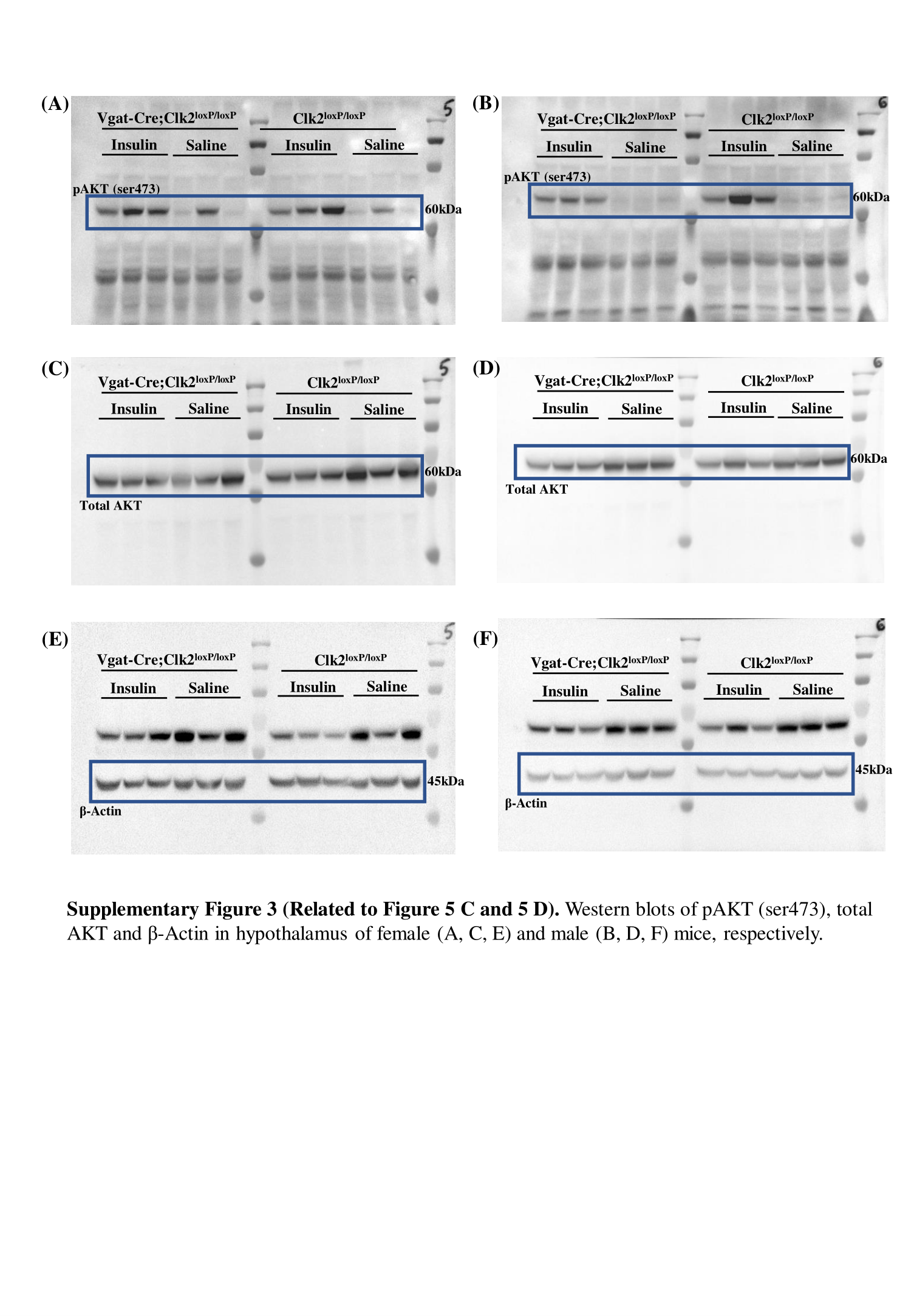

Supplement: Supplementary file 5 [file Image_3.tiff]
